# Remarkable Magnetic Performances and Thermal Analysis of Nanocomposite Melt-Spun MnAl(Ga) Alloys

**DOI:** 10.3390/nano16181177

**Published:** 2026-09-17

**Authors:** Alina Daniela Crisan, Cristina Bartha, Gabriel Alexandru Schinteie, Ovidiu Crisan

**Affiliations:** National Institute for Materials Physics, 077125 Magurele, Ilfov, Romania

**Keywords:** L1_0_ phase, rare-earth-free magnets, thermal analysis

## Abstract

Several potential applications of rare-earth-free magnets have been identified in emerging technological domains such as renewable energy and e-mobility in smart cities, with emphasis on electric bikes and scooters, as well as in the field of autonomous vehicles. In all these applications, with operability at high temperatures, there is considerable interest in nanocomposite magnetic alloys based on MnAl binary systems. The reason for such interest is represented by the occurrence of the τ-MnAl phase, which is magnetic and structurally compatible with L1_0_ tetragonal phases. While the research on MnAl and MnAl-derived magnets has been extensive, alloys with the addition of post-transitional metals with an orthorhombic structure, such as Ga, are less often investigated. The purpose of such additions is to promote and preserve the formation and stability of the τ-MnAl phase, which has promising magnetic properties. The paper illustrates the thermal stability and optimized magnetic properties of τ-MnAl in an alloy based on an off-equiatomic MnAl system with 4 at% Ga addition. A thorough thermal analysis involving a differential scanning calorimetry study is reported, powered by kinetic analysis using two complementary iso-conversional methods: Friedman and Ozawa–Flynn–Wall. Structural characterization is performed using XRD, with the results supported through full-profile MAUD analysis (version 2.99, University of Trento, Italy), revealing that the hexagonal ε phase, predominant in the as-cast sample, transforms massively into the tetragonal τ phase, which becomes predominant upon annealing. Magnetic measurements are employed to fully characterize the alloy’s magnetic properties. It is shown that the phase transformation creates very good conditions for achieving good remanence values of about 210 kA/m and large intrinsic coercivities of about 446 kA/m at ambient temperatures.

## 1. Introduction

In recent years, research in the field of rare-earth-free magnetic materials for various uses has focused on the discovery and development of magnet systems with improved stability, properties and performance. This has often been accompanied by the scientific and technological challenge of progressively increasing intrinsic and extrinsic magnetic properties while understanding the fundamental mechanisms behind these properties. Magnetic materials are globally applicable in practically all industrial sectors, such as information technologies, medicine, and renewable energies, as well as in the automotive and e-mobility industry, particularly in devices for autonomous electric and hybrid vehicles or wind turbine generators.

The research community has intensively investigated new magnetic materials that either do not contain rare earths or contain a strongly reduced content of rare earths. In particular, rare-earth-free nanocomposite magnets are actively researched, and, in this category, metallic alloys based on low-cost elements such as Mn and Al have been investigated the most [1,2,3,4,5,6]. There is another reason for this high interest, namely, their applicability in extreme conditions of operation. As their Curie temperature is higher than that of classic permanent magnets, such magnets can operate in applications requiring high temperatures. Many systems of magnetic materials not containing rare earths have been investigated, most commonly in intermetallic compound systems, as reported for FePt [7], MnAl [8] or MnBi [9].

The common ground for all these alloys is that in certain instances, they crystallize into the tetragonal L1_0_ phase. An exception is MnBi, whose ferromagnetic low-temperature phase (LTP) adopts a hexagonal structure (P63/mmc). This crystal structure represents a high-order distortion of a cubic precursor, which was proven to exhibit good magnetic anisotropy as well as strong coercive fields [7,8]. Until now, the most intensive research efforts were devoted to L1_0_ FePt. However, there has been a recent surge of interest in compounds with lower costs, such as those based on Mn and Al. These are interesting for mainly two reasons: the low cost of components and the versatility of non-equilibrium synthesis techniques for producing bulk alloys with the potential of obtaining L1_0_-type magnetic phases. It is clear that chemical production routes are not suitable for yielding large amounts of magnets in quantities that are relevant for industrial use. For this purpose, synthesis paths for bulk materials, such as the melt spinning technique, are deemed suitable for RE-free alloys [10,11]. The melt spinning technique is appropriate for solid-state magnets exhibiting the L1_0_ tetragonal phase or the τ phase, as found in MnAl-type alloys [12] that are also suitable for other RE-free magnetic materials, such as MnGa. Since there are other competing phases in MnGa, susceptible to formation after annealing, a non-equilibrium synthesis technique is required to obtain thermodynamically stable ferromagnetic phases. This is also the case in MnAl binary alloys [13]. It was shown [13] that appropriate annealing induces transformation of rapidly solidified MnAl ribbons from the as-quenched metastable antiferromagnetic ε-phase to the ferromagnetic *L*1_0_ τ-phase of interest. This phase has valuable intrinsic magnetic properties, saturation magnetization Ms > 0.7 MA/m, magnetic anisotropy K_1_ > 1.5 MJ/m^3^, theoretical maximum energy product of 120 kJ/m^3^, and Curie temperature T_C_ > 600 K. This is the only ferromagnetic phase that occurs in the Mn-Al phase diagram, having an ordered face-centered tetragonal structure (fct) of P4/mmm symmetry. Such an L1_0_ phase cannot be obtained in alloys with stoichiometric composition, but it is formed as a metastable state in a narrow range of the phase diagram, with non-equiatomic composition Mn_50+x_Al_50−x_. Various attempts have been made to stabilize this structure, for instance, by adding a few at% of Bi and C elements, but only a few studies regarding the thermal stability of these phases have been published until now [14]. On the other hand, the MnGa phase diagram [15] exhibits a few magnetically different phases in the vicinity of 75 at% Mn. One stable phase is the hexagonal D0_19_ phase Mn_3_Ga, which is antiferromagnetic and possesses non-collinear magnetic structures [16]. A competing structural allotropic phase in this compositional range is the tetragonal D0_22_ phase Mn_3_Ga, which is a ferrimagnet [17,18]. A different concurrent structure in this range of stoichiometry is the tetragonal L1_0_ MnGa. As is the case with other L1_0_ phases, it is a hard magnet with high magnetic anisotropy, large coercivity and high magnetization [19]. This peculiar combination of magnetic phases was reported to be due to the electronic structure of half-filled *d* levels of Mn atoms [20]. In our previous work [21], we showed that in some binary MnGa alloys, after annealing at 400 °C and 500 °C, a multi-phase microstructure is obtained, where crystallites of both L1_0_ and D0_22_ tetragonal phases co-exist. Due to the interplay between these magnetic phases, which are structurally and magnetically different, promising magnetic performances were obtained, namely, coercivity and remanence surpassing previously reported values in bulk alloys and thin films of MnGa.

A step towards increased magnetic performance could be doping the MnAl binary system with Ga to obtain the optimal ferromagnetic *L*1_0_ τ-phase of interest, MnAl, thermally stabilized by adding Ga whilst taking advantage of the good magnetic properties also obtained in MnGa, where both the L1_0_ and D0_22_ tetragonal phases may co-exist. In that sense, several previous works showed good perspectives for improvements in the thermal stability of the phase structure in MnAl doped with Ga. For instance, in [22], it was shown that the magnetic properties, phase and structural state of Ga-doped τ-MnAl during ball-milling show thermal stability, but after annealing, τ-MnAl decomposes and coercivity is then diminished. In another work [23], it is shown that in some ternary alloys of stoichiometry Mn_55_Al_45−x_Ga_x_ with 5 < x ≤ 9, after appropriate heat treatments, two different phases with the L1_0_ structure can co-exist. Also, some progress can be made by carbon addition, as shown in [24], where magnetic properties of (Mn_54_Al_46_)_98_C_2_ magnets made through liquid-phase sintering with Mn_65_Ga_35_ as an additive can be optimized. From a theoretical point of view [25], it was shown that substitutional atoms of Ga improve the intrinsic magnetic properties in L1_0_ MnAl alloys by annealing according to ternary phase diagrams.

All these aspects motivate us to study the thermal stability of a MnAl binary alloy doped with Ga, which is needed to optimize the material phase structure and phase compositions for a stable tetragonal phase during annealing and enhanced magnetic performance.

This paper presents a study of an alloy based on an off-equiatomic MnAl system with 4 at% Ga addition. Thorough thermal analysis illustrates its thermal stability and paves the way to demonstrating optimized magnetic properties of the τ-MnAl phase.

The paper’s aim is to contextualize our prior research on MnAl, MnGa, and other intermetallic systems [8,12,14,21]. Based on our earlier findings, which established good performance, as well as thermal and magnetic baselines for MnAl nanocomposites and, moreover, demonstrated the potential of MnGa materials, the primary goal of this work is to extend those findings by examining the specific influence of Ga-doping on crystallization kinetics and the phase transformation mechanisms of the MnAl system. The proven phase transformation creates very good conditions for achieving high remanence values and large intrinsic coercivities at ambient temperature. This proves that among other L1_0_-based magnetic materials, systems based on MnAl are promising as performant and cost-effective magnets in several industrial applications.

## 2. Materials and Methods

The synthesis procedure, i.e., ultra-rapid solidification from the melt, involves a Buehler melt spinning system (Buehler Melt Spinner MSP 10 from Edmund Buehler GmbH, Bodelshausen, Germany). For the synthesis of the initial alloy, Mn, Al and Ga metal flakes with a high purity of 99.99% from Merck (Merck KGaA, Darmstadt, Germany) are used. Pre-alloying of the precursor is performed by melting in an arc oven, in a controlled argon atmosphere, at a low pressure of 10^−1^ Torr. The obtained pre-alloys of nominal composition, i.e., Mn—53 at%, Al—43 at% and Ga—4 at%, are then re-melted no less than three times in the same oven. This ensures a high degree of homogeneity in the alloy and a complete alloying of the initial elements. In the next step of the synthesis procedure, a quantity of 5 g of each pre-alloy is introduced into a quartz crucible mounted inside the melt spinner. The crucible is mounted on top of the rotating wheel inside the melt spinner chamber. It is also inductively heated to bring the pre-alloy to the molten state. There is a 3 mm nozzle at the bottom of the crucible in close proximity to the rotating copper wheel. By the time the pre-alloy is completely melted inside the crucible, an argon overpressure of 50 kPa is applied inside the crucible. The immediate consequence is that the melt is completely purged onto the surface of the rotating copper wheel. The rotation frequency of the copper wheel is set to roughly 1150 rot/min, which is equivalent to a peripheral velocity of about 26 m/s. When in contact with the fast-rotating copper wheel, the melt solidifies almost instantly with a cooling rate of about 10^5^ K/min, giving rise to continuous ribbons more than 5 decimeters long, 2.5 mm wide and about 45 microns thick.

The as-cast ribbons are thermally annealed further to ensure the formation of tetragonal L1_0_ phases. For this purpose, we use an isothermal annealing procedure for a duration of 1 h at 800 °C. The annealing procedure is performed in a controlled-atmosphere resistive oven under a low vacuum of 10^−3^ Torr.

The thermal behavior and phase evolution of Mn_53_Al_43_Ga_4_ with temperature were investigated by the differential scanning calorimetry technique using a Netzsch STA 449 F3 Jupiter from Netzsch GmbH, Selb, Germany, with a simultaneous thermal analyzer in TG-DSC mode. The samples, each weighing 30 mg, were measured in an inert argon atmosphere at heating rates of 5, 10, 15, 20, and 25 °C min^−1^, from room temperature up to 800 °C. The experiments were conducted in open cylindrical alumina crucibles at a gas flow rate of 20 mL min^−1^. The heat flow precision was ± 0.001 mW, and the temperature accuracy was ± 0.01 °C. A non-isothermal kinetic analysis of the DSC data was performed using Netzsch Thermokinetics 3.1 software (version 072010).

The kinetic parameters of the crystallization process were determined comparatively using two model-free approaches: Friedman and Ozawa–Flynn–Wall models. Both are iso-conversion techniques for analyzing the kinetics of reactions in DSC curves and allow the determination of activation energy (*E*_a_) and the pre-exponential factor (log A) without assuming a specific kinetic model for the crystallization process [26]. Theoretical studies have revealed that the equation that describes the relationship between the reaction rate, reacted fraction, and temperature, independent of the thermal sequence used for experiments, has the following form:

$$\frac{dx}{dt}=Aexp(\frac{-Ea}{RT})f(x)$$

where *x* represents the reacted fraction, *A* is the Arrhenius pre-exponential factor, *R* is the gas constant, *E*_a_ is the activation energy, *f*(*x*) is the kinetic model (which is constant in the model-free analysis), and *T* is the temperature of the process [27]. The Friedman model is a differential method that plots the logarithm of the reaction rate (ln(d*x*/d*t*)) against 1/*T* for measurements at different heating rates (*β*), while the Ozawa method is an integral method that plots the logarithm of the heating rate (log *β*) against the inverse temperature at a constant conversion (*α*) [28]. Both models are usually used together to verify the consistency of the calculated kinetic parameters (*E*a and Log A), although there are some differences in their sensitivities. When the activation energy remains constant throughout the conversion process, both methods generally lead to similar results. However, if the activation energy varies with the conversion, as is the case in complex reactions, the two methods may provide different values for the activation energy [29]. Following these analyses, the crystalline phases formed during heat treatment were studied structurally and identified by X-ray diffraction studies.

The structural characterization was performed through X-ray diffractometry using a D8 Advance diffractometer (Bruker AXS GmbH, Karlsruhe, Germany). X-ray diffractograms were obtained in powder diffraction mode, under ambient conditions and a θ–2θ incidence geometry. The angular interval of analysis was between 20° and 80° (in 2θ), and the X-ray beam used was Mo Kα1 radiation with a wavelength λ = 0.71 Å. For full-profile analysis of the obtained diffractograms, a Rietveld-type refinement analysis was performed on all the obtained XRD patterns. The analysis involved the use of MAUD (Materials Analysis Using Diffraction) software (MAUD version 2.99, University of Trento, Italy).

All magnetic measurements, such as hysteresis loops and specific magnetization measurements, were performed utilizing the vibrating sample magnetometry module of an MPMS (Magnetic Properties Measurement System) from Quantum Design (Quantum Design Europe GmbH, Darmstadt, Germany). This MPMS device is extremely sensitive, being capable of detecting magnetic moments as low as 10^−8^ emu. It provides up to 10^−11^ A m^2^ resolution and up to 12 T applied magnetic field over a temperature range between 2 and 400 K. The magnetic determinations were carried out on both as-cast and annealed samples in an applied field of up to 12 T, which was set parallel to the ribbon plane. All measurements were performed between 10 K and 300 K.

## 3. Results and Discussion

### 3.1. Structural Characterization

#### 3.1.1. Differential Scanning Calorimetry

**Figure 1:**
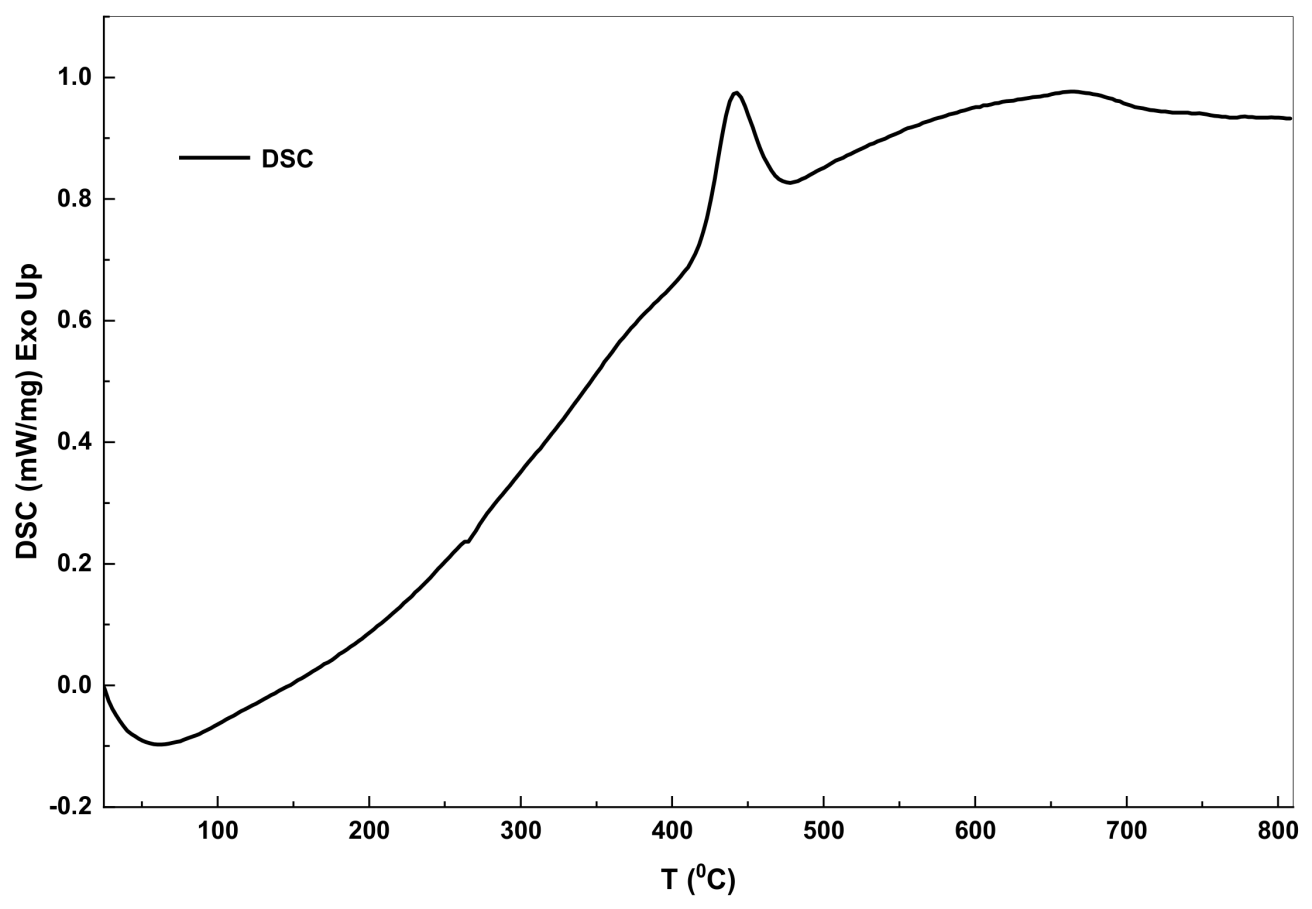
Differential scanning calorimetry for Mn_53_Al_43_Ga_4_ sample recorded at a rate of 5 °C/min.

**Figure 2:**
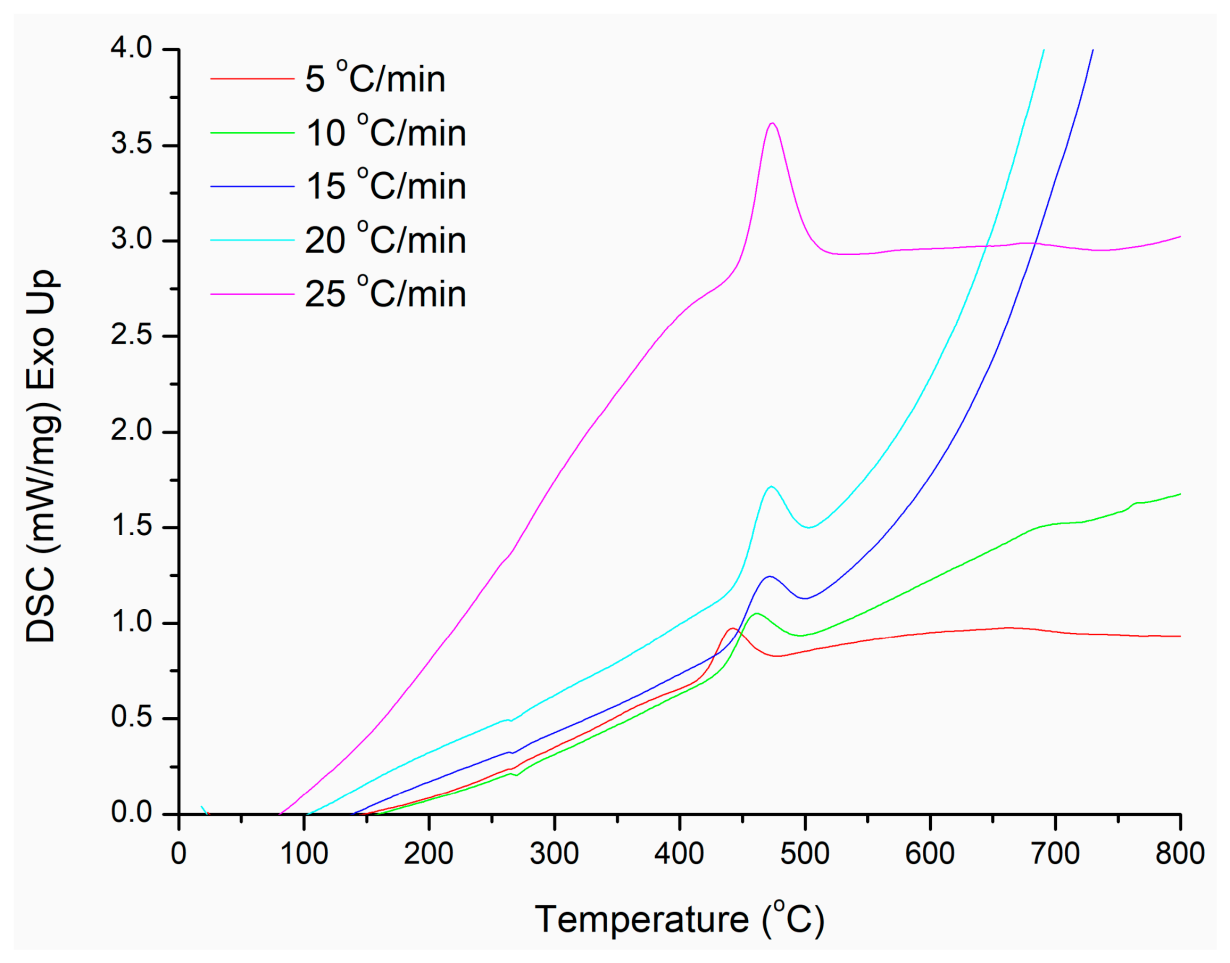
The crystallization peaks for Mn_53_Al_43_Ga_4_ samples, recorded at heating rates from 5 °C/min to 25 °C/min.

For the Mn_53_Al_43_Ga_4_ sample, the DSC scan at 5 °C/min is shown in Figure 1. All the DSC scans recorded at the five different heating rates are comparatively presented in Figure 2. The DSC curve shows a pronounced exothermic peak that begins around 420 °C and reaches its maximum intensity around 460 °C. This peak is characteristic of the crystallization process occurring within the sample. This peak starts around 420, with a maximum intensity around 460 °C. The onsets of the crystallization peaks (420–460 °C) are dependent on the heating rate, with a central maximum located in the range between 442 and 474 °C (Figure 2).

The maximum temperatures of each of the recorded peaks are listed in Table 1.

It can be observed that all T_peak_ values increase with increasing heating rate and shift towards higher temperatures, which implies a slower heat transfer. This, in turn, suggests that a higher transition temperature is required for structural arrangements and that higher crystallization temperatures are required to allow the particles to be rearranged into ordered structures. Below, we describe the crystallization kinetics analysis under non-isothermal conditions for the Mn_53_Al_43_Ga_4_ sample.

**Figure 3:**
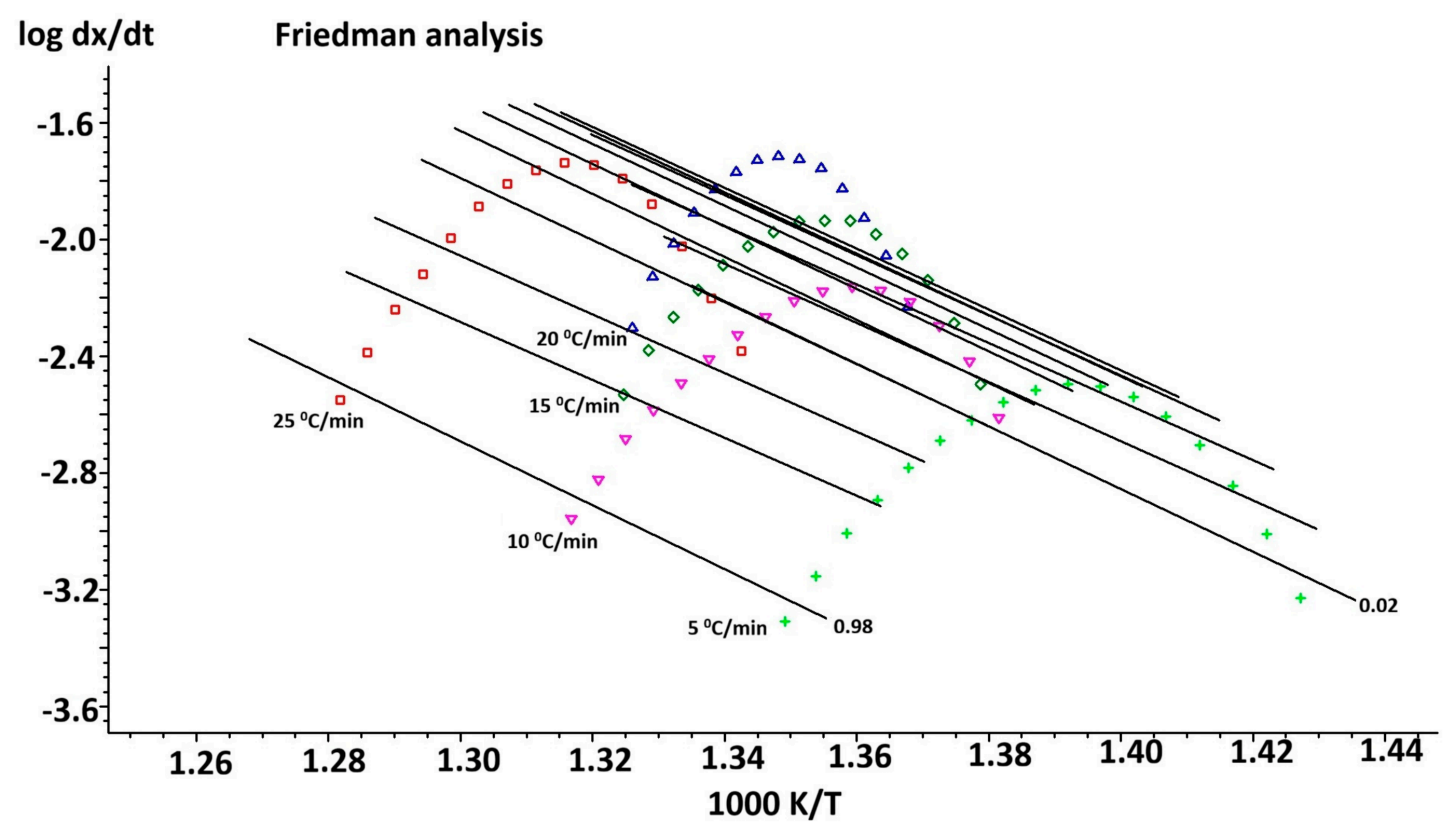
Friedman analysis of the Mn_53_Al_43_Ga_4_ sample.

**Figure 4:**
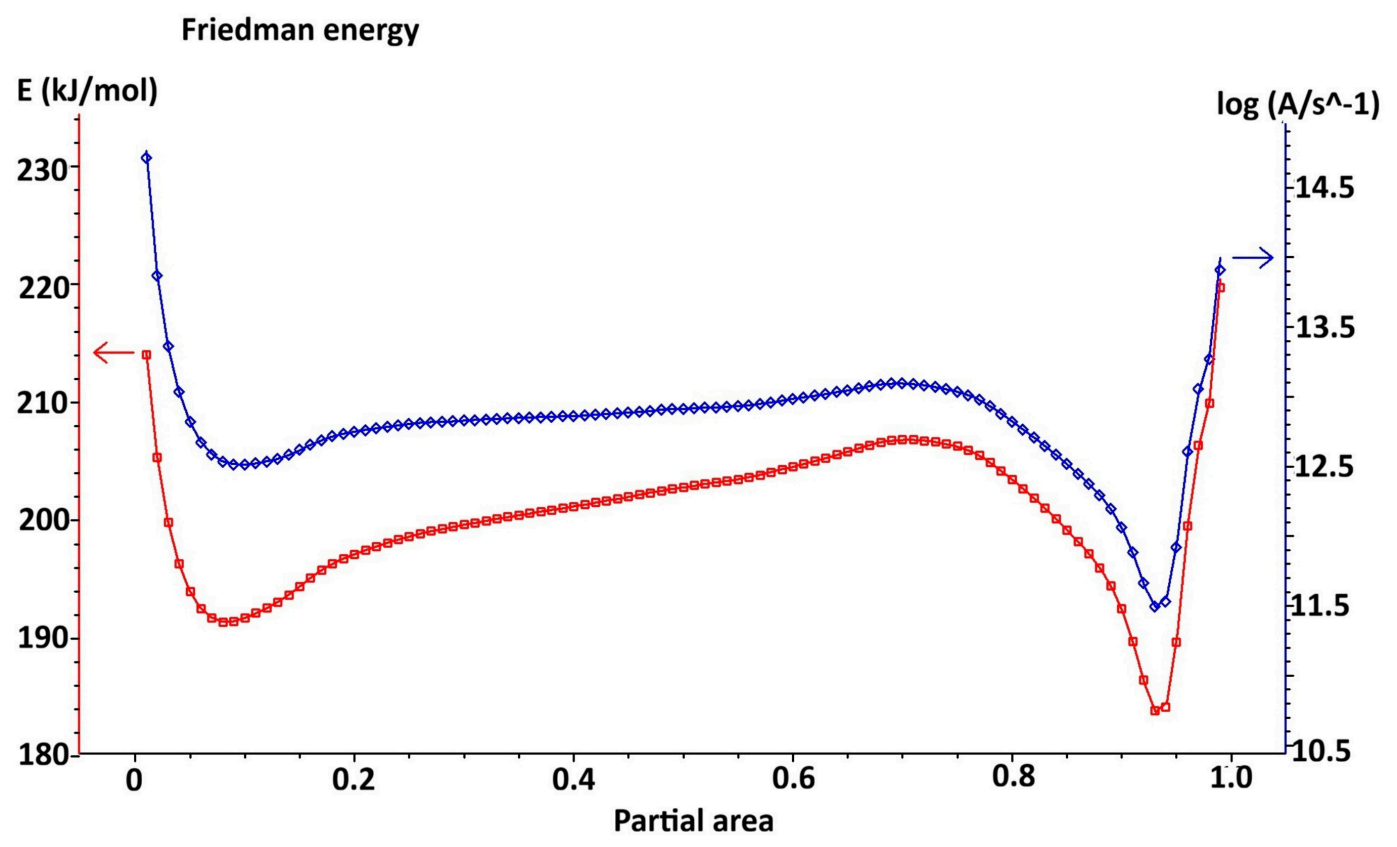
Friedman activation energy of the Mn_53_Al_43_Ga_4_ sample.

The activation energy of the crystallization process was comparatively analyzed by the two model estimates described above. From the analysis of the crystallization peak, it can be observed that both the onset and offset of the transformation show different levels of heat flux, which indicates that the crystal phases at the beginning and end of crystallization are not identical. This is confirmed by X-ray diffraction, where the presence of phases with different structures and symmetries is observed, which is studied hereafter. It can be seen that all the characteristic crystallization peaks are wide, with low intensities for the first two heating rates, indicating that a smaller fraction of the sample is undergoing a crystallization process. In the case of higher rates of heating, higher (more intense) peaks are observed, which implies the crystallization of a major fraction of the sample. Figure 3 and Figure 4 show Friedman’s analysis of the DSC data of the Mn_53_Al_43_Ga_4_ sample measured under distinct heating rates, as well as Friedman’s activation energies. The graph in Figure 3 shows the dependence of the log value of dx/dT vs. 1000/T, i.e., the crystallization rates at certain values of x (x is the crystallization fraction). A series of straight lines (iso-conversional lines) are obtained for each heating rate for a certain fraction of crystallization. The straight lines are iso-conversional lines, i.e., data points from multiple DSC curves measured at different heating rates, sampled at identical conversion levels (α) and connected to form linear fit lines. As the slope depends on the crystallization fraction, it indicates how the activation energy changes during the transformation reaction.

In Figure 4, the right vertical axis represents Log (A/s^−1^). In the Friedman iso-conversional method for kinetic analysis, A or A_α_ is the pre-exponential factor (or frequency factor) derived from the Arrhenius equation. It relates to the frequency of molecular collisions or transition-state entropy at a specific degree of conversion α. Log(A/s^−1^) represents the decadic logarithm of the pre-exponential factor (or frequency factor) A expressed in reciprocal seconds s^−1^. As shown in Figure 4, the maximum activation energy for this crystallization peak is obtained for a conversion rate of 75% (207.98 kJ/mol). From the energy graph, we determine that the crystallization process occurs as a result of a single reaction because the *Ea* curve shows no variation up to a fraction of 75% when the crystallization process is almost complete. The activation energy increases almost constantly with the increase in the fraction of crystallized volume, followed by a decrease. Based on this result, it can be predicted that the crystallization process is facilitated by the annealing duration as well as by the increase in the crystallized volume fractions. This is because the energy required for nucleation in the initial stage decreases continuously as the crystallization process progresses, and the energy is used solely for the growth process.

**Figure 5:**
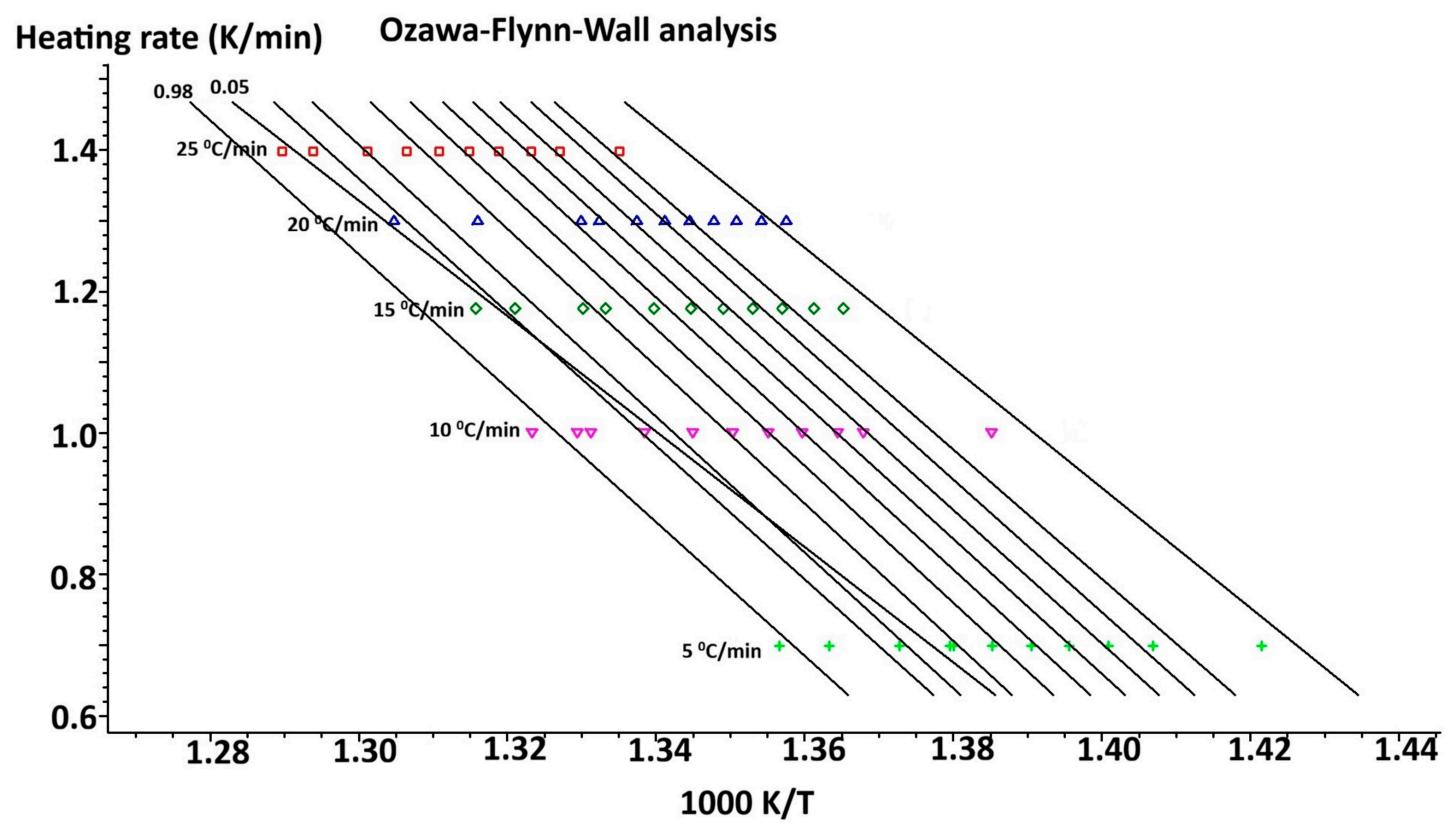
Ozawa–Flynn–Wall thermal analysis for the Mn_53_Al_43_Ga_4_ sample.

**Figure 6:**
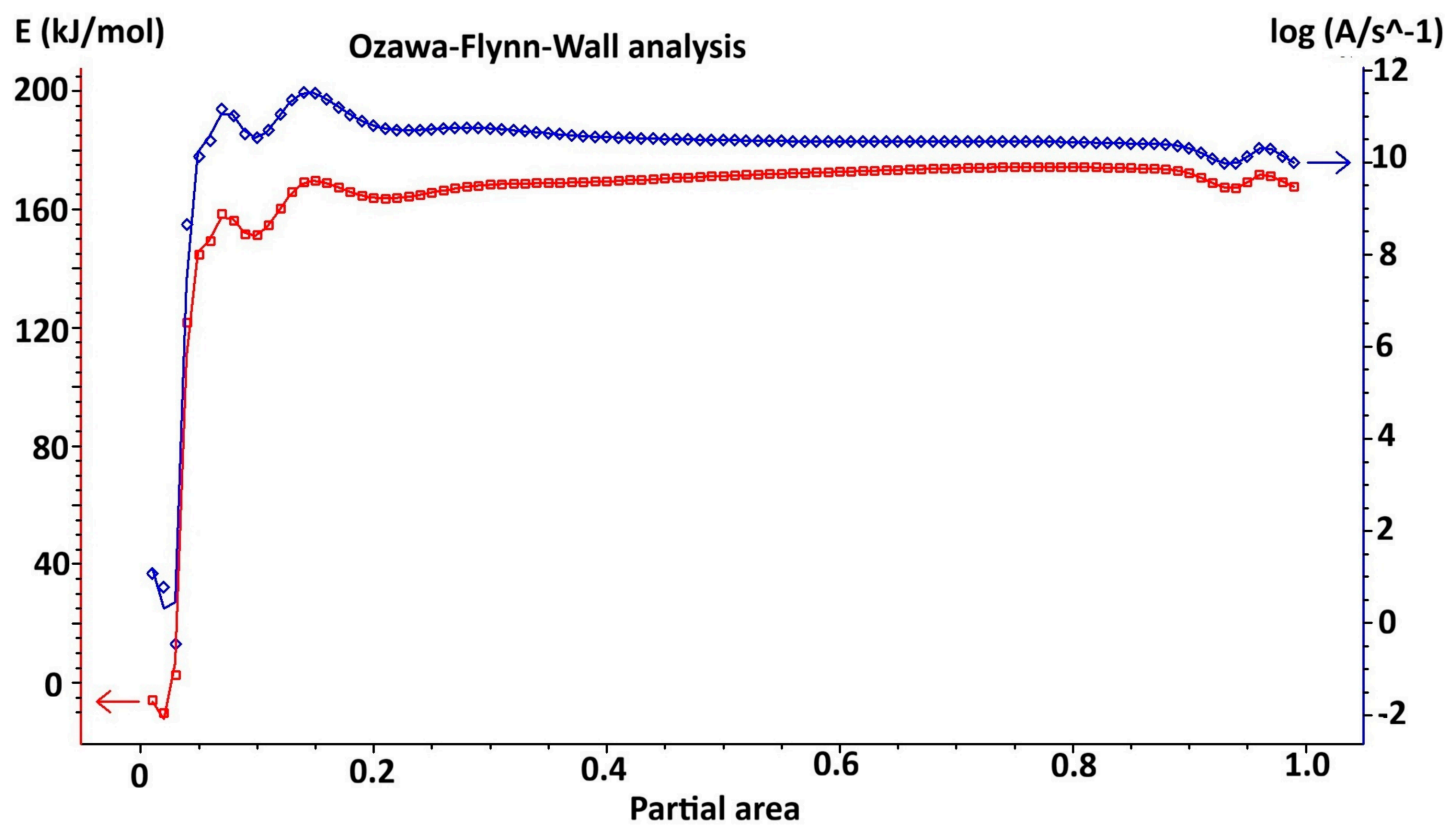
Ozawa–Flynn–Wall activation energy analysis for the Mn_53_Al_43_Ga_4_ sample.

The second kinetic evaluation obtained by applying the Ozawa–Flynn–Wall free model is shown in Figure 5, and the activation energy values are represented in Figure 6. Maximum activation energy values are obtained for two conversion rates. The first value is 168.49 kJ/mol for a 20% conversion rate, and the second value is 169.30 kJ/mol for a 95% conversion rate. It is observed that after the initial maximum value of the activation energy, the dependence remains almost constant until a crystallization fraction of 85%, which means that the crystallization process becomes slower. In this case, the decrease in activation energy for a fraction of 90% crystallization is slower than in Friedman’s model. The observed decrease in activation energy with temperature demonstrates that the rate of crystallization is actually determined by the rates of two processes: namely, nucleation and diffusion. A decrease in the apparent activation energy of crystallization with rising temperature reveals that the overall process combines a thermodynamically driven nucleation barrier and a temperature-dependent molecular diffusion step. As the temperature changes, the shifting balance between the free energy required to form critical nuclei and the mobility of diffusing atoms alters the effective kinetic resistance. The effective activation energy declines with temperature, proving that neither thermodynamics (nucleation alone) nor mass transport (diffusion alone) entirely dominates the heating rate; instead, the overall speed relies on both working in tandem. Following both evaluations, the *Ea* values were determined to be within the accepted limits (±50 KJ/mol [29]). The comparative kinetics of the processes identified in the sample are very important, as it provides essential information on the transformation mechanisms through two parameters (activation energy and pre-exponential factor). In our case, the values of the activation energies calculated by the methods mentioned above are close to each other. The pre-exponential factor, also known as the frequency factor, provides information about the number of attempts, within a unit of time, made by nuclei to overcome the energy barrier and commence nucleation. In addition, it also indicates the number of nucleation sites and has a downward trend. At higher temperatures, a decrease in activation energy values and pre-exponential factors is observed, which means that the crystallization process is almost completely terminated. This behavior can be explained by the existence of two processes in this stage of crystallization: the first is structural transformation and the second is the continuous growth of crystallites.

#### 3.1.2. X-Ray Diffraction Analysis and Characterization

Structural characterization was performed by X-ray diffraction using Mo Kα1 wavelength radiation (wavelength 0.71 Å) and a LiF crystal monochromator in a Bragg–Brentano diffraction geometry. Diffractograms were obtained at room temperature, in an analysis range from 20° to 80° (in 2θ), with a step scan interval of 0.02° and a dwell time of 10 s per step. The use of Molybdenum Kα1 radiation for the diffraction study is justified by its short wavelength (0.71 Å) and high energy (17.44 keV for Kα), which allows for a greater depth of penetration due to its high atomic number and is useful for the analysis of denser or thicker samples.

**Figure 7:**
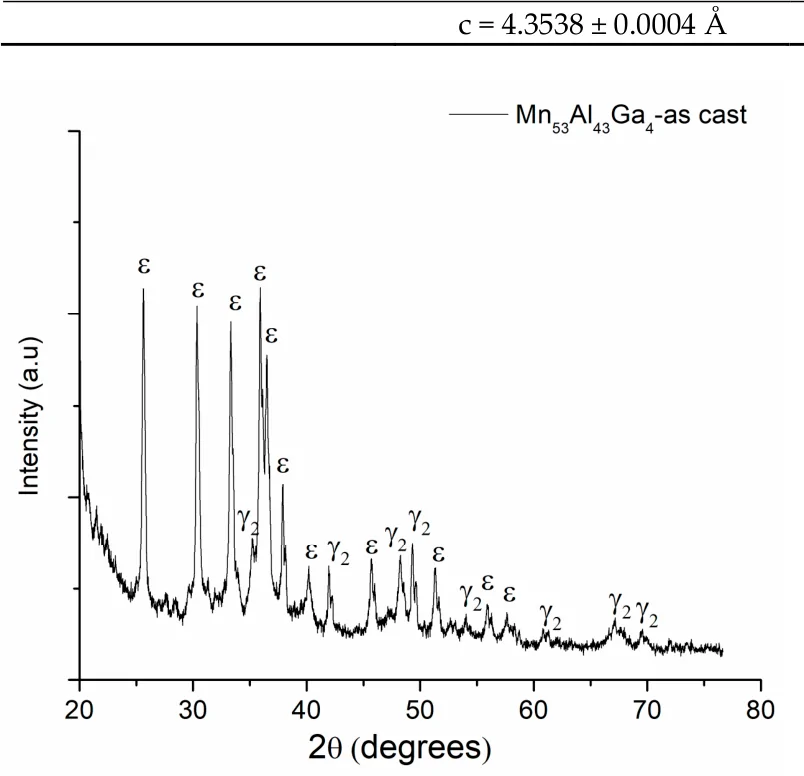
XRD for the Mn_53_Al_43_Ga_4_ as-cast sample.

The diffractogram for the Mn_53_Al_43_Ga_4_ sample is shown in Figure 7. The diffraction of the as-cast sample of the ternary alloy Mn_53_Al_43_Ga_4_ shows characteristics that are typical of polycrystalline materials, with a multitude of narrow diffraction lines characteristic of metallic systems with high crystallinity. The diffractogram was fitted with full-profile MAUD software, and the results of the fitting are summarized in Table 2. In the diffraction data, the diffraction lines are indexed as belonging to the ε crystalline phase (hexagonal symmetry, space group *P6/*mmm, ICDD: 01-074-5244). The diffraction lines belonging to the ε phase are visible and easily identifiable. Some other diffraction lines of lower intensity are also observable. After analyzing the diffraction data, we attribute these lines of lower intensity to the γ2 rhombohedral symmetry phase (space group *R3m*, ICDD 00-031-0021). Following the phase analysis, we were able to index all diffraction lines, and it was observed that the crystal phase with a higher degree of order, ε, is predominant, along with diffraction lines corresponding to the γ_2_ phase. The full-profile MAUD analysis revealed that the relative abundance of the hexagonal phase ε is very high, at about 94%.

In the ternary sample, the crystallite size is of the order of a few tens of nanometers. This clearly indicates the important role played by the addition of Ga atoms, namely, the structural refinement of the constituent cell unit of the phases and the realization of truly nanometric structures, an important aspect in optimizing the properties of alloys.

**Figure 8:**
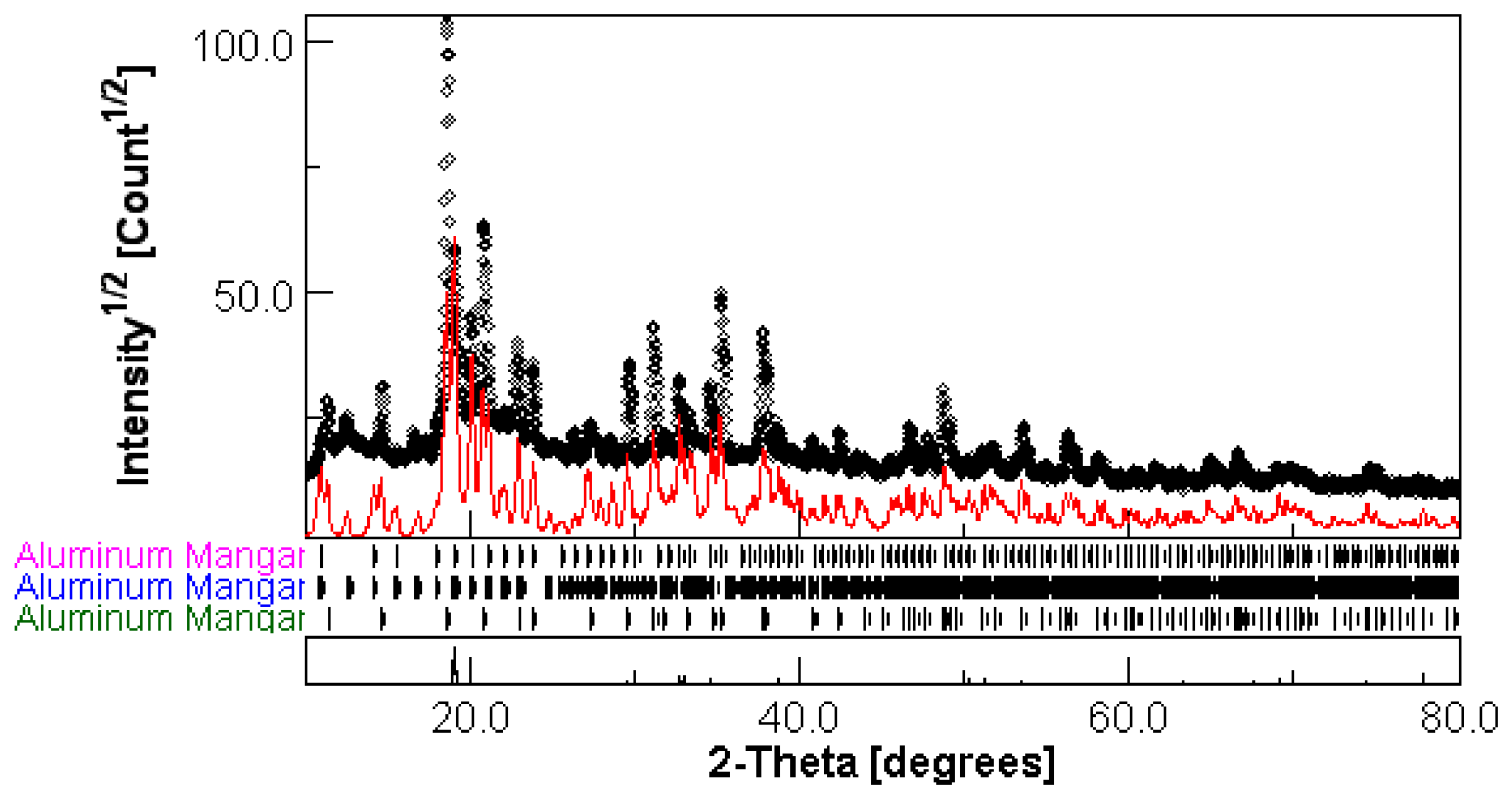
Full-profile MAUD analysis of the diffractogram for the Mn_53_Al_43_Ga_4_ annealed sample. The indexing lines are color-coded: pink for the rhombohedral phase, blue for the hexagonal phase and green for the tetragonal phase.

To transform the hexagonal phase into the tetragonal τ (or L1_0_) phase, which is a highly desired phase due to its high magnetic performance, isothermal annealing for 1 h was performed on the Mn_53_Al_43_Ga_4_ as-cast sample. The isothermal annealing temperature was set to 800 °C. This value was chosen taking into account the crystallization peak position as well as the positions of the other small kinks observed in the DSC curves at about 700 °C. We chose annealing at 800 °C, the maximum investigated temperature in DSC studies, to go beyond the last small kinks observed. The resulting annealed sample was analyzed through X-ray diffraction, and the resulting diffractogram, fitted with the full-profile MAUD analysis, is shown in Figure 8.

The full-profile fitting of the diffractogram for the annealed sample revealed, apart from the presence of the ε-phase of hexagonal symmetry and the small rhombohedral γ2-phase, the occurrence of a third phase, resulting from the structural phase transformation of the ε-phase into the tetragonal τ-phase (space group P4/mmm, ICDD: 04-026-5412). The results of the full-profile fitting of the annealed samples, relative abundance, lattice parameters and average grain size are given in Table 3.

It is seen that after annealing under the conditions dictated by the results of the thermal analysis, there is a massive structural phase transformation, where almost 2/3 of the hexagonal phase undergoes an ordering phase transition with the occurrence of a predominant (71%) tetragonal τ-phase in our alloy. This is a prerequisite for obtaining significant magnetic properties in the annealed sample, as revealed in the following section.

### 3.2. Magnetic Characterization

To obtain a more elaborate image and investigate the magnetic properties more deeply, several magnetic measurements were performed on the annealed sample. These were meant to pinpoint the influence that the crystallographic phase structure has on the magnetic properties of the annealed sample. These measurements were performed by SQUID magnetometry at temperatures ranging from 10 K to 300 K. More specifically, studies on magnetization as a function of temperature were performed, as well as hysteresis loops that were recorded in a parallel configuration, parallel to the ribbon’s plane. The hysteresis loops were recorded at various temperatures; two of them, at 10 K and 300 K, are presented hereafter.

**Figure 9:**
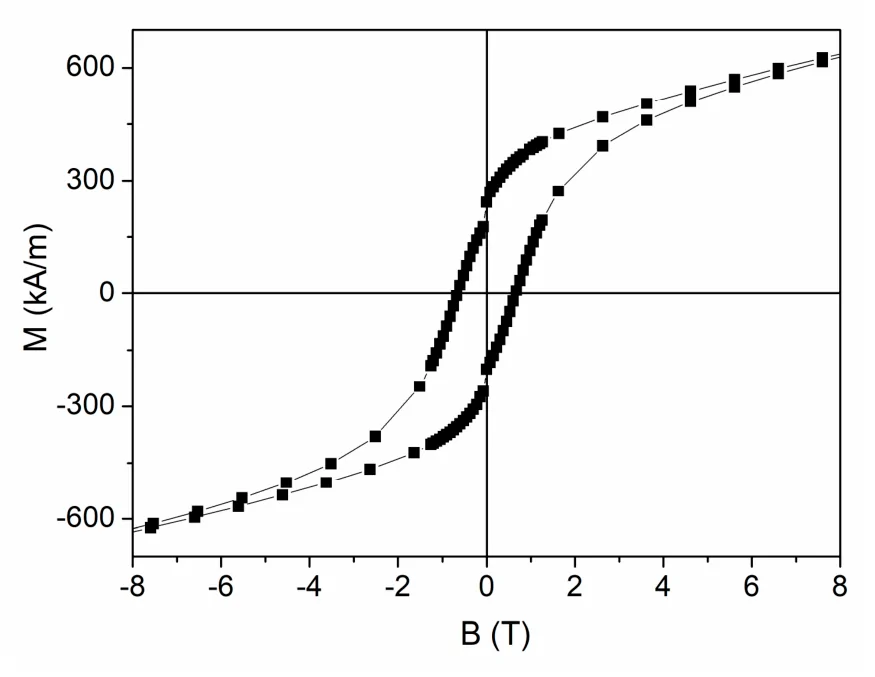
Hysteresis loop for the annealed sample at 10 K.

**Figure 10:**
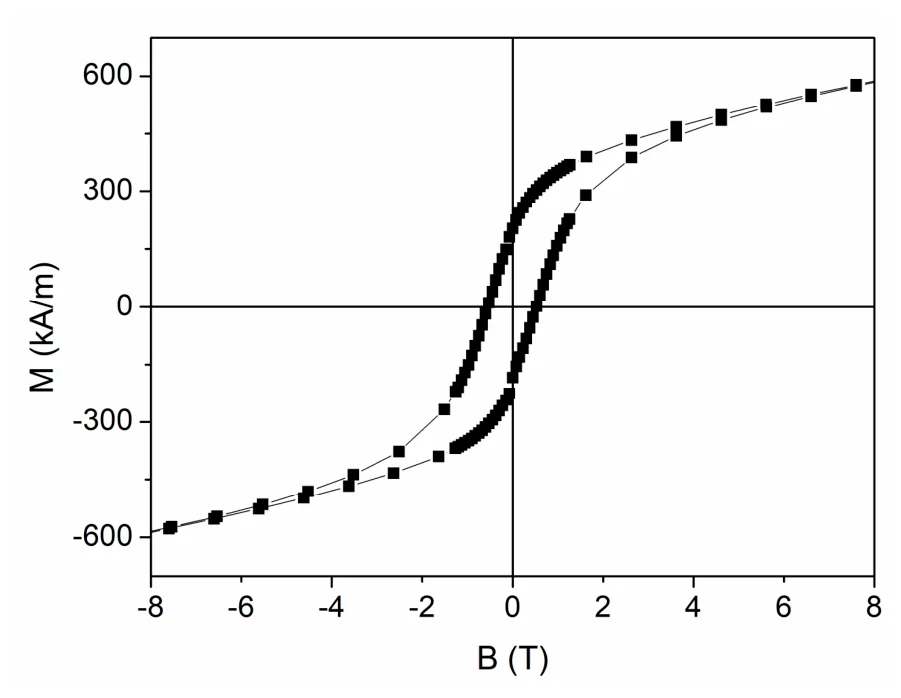
Hysteresis loop for the annealed sample at 300 K.

Figure 9 and Figure 10 present the hysteresis loops for the annealed sample recorded in an applied magnetic field of 5 T, at 10 K and 300 K, respectively.

At both 10 K and 300 K, the annealed sample exhibits features that are typical of hard magnetic materials. More specifically, the curves are characteristic of ferromagnets, and they show large remanence and high coercivity for both the 10 K hysteresis loop (252 kA/m; 0.68 T in terms of magnetic flux density or 541 kA/m in terms of field strength) and the 300 K hysteresis loop (210 kA/m; 0.56 T in terms of magnetic flux density or 446 kA/m in terms of field strength). The reason for such strong performance in the studied alloy is linked to the successful structural phase transformation, optimized and monitored through the kinetics models that allowed us to analyze the activation energy of the transformation. Therefore, there is a direct correlation between magnetic performance (remanence/coercivity) and successful transformation to the tetragonal τ-phase, which is the cause and main reason for such high values.

**Figure 11:**
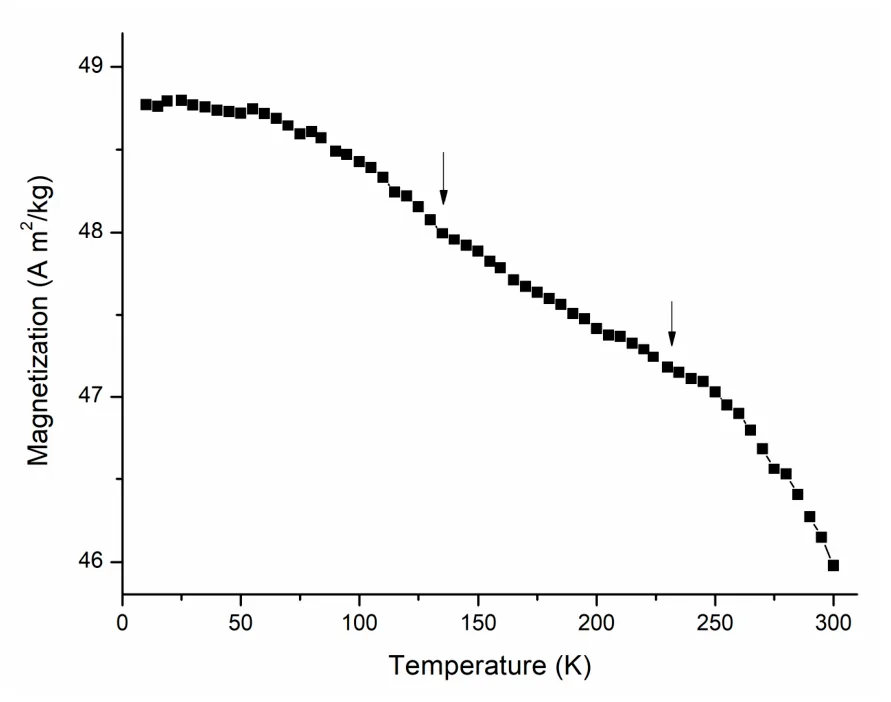
Magnetization as a function of temperature for the annealed Mn_53_Al_43_Ga_4_ sample.

Figure 11 shows the magnetization versus temperature for the annealed sample. The measurement was taken under an applied field of 100 Oe. The obtained M vs. T curve is specific to a typical 3D Heisenberg ferromagnet, with magnetization diminishing from low to high temperatures. It is worth mentioning that good magnetic performances are also obtained at the maximum temperature in the study (300 K). Furthermore, the M vs. T dependence shows two very weak inflection points, at about 134 K and 231 K (illustrated by black arrows). This change in slope potentially illustrates that the temperature dependence of the magnetization changes due to the other two phases that are still present in the annealed samples in lower abundances, namely, the hexagonal ε and the rhombohedral γ2 phases, which have different ordering temperatures.

These remarkably good magnetic performances are facilitated by the phase structure of the annealed sample, where the hexagonal phase ε, obtained in an overwhelming proportion in the as-cast state, is strongly diminished in the annealed samples where the tetragonal τ-phase is predominant, co-existing with the remaining hexagonal phase and traces of rhombohedral MnAl phases. The conditions of the ε → τ transformation are dictated by the composition of the binary alloy and the doping with additional phase-stabilizing elements. τ-MnAl is formed from the high-temperature hexagonal precursor phase ε-MnAl upon annealing at approximately 500 °C. However, the ε → τ transformation is not yet complete at this temperature; hence, there is a need to go to higher temperatures that are close to the sintering conditions for producing compact magnets for real-life applications. The ε—MnAl phase is magnetically an antiferromagnetic phase, with a Néel T_N_ temperature of 90 K, and the L1_0_ phase (τ) tends to decompose into a solid solution of the γ2 and β-(Mn) phases at temperatures above 650 °C, according to the Mn-Al phase diagram. To prevent this shortcoming, the binary alloy can be doped with various impurities; hence, the role of adding Ga. In the tetragonal phase of MnAl, Ga atoms enter the lattice by directly substituting Al atoms, occupying the corresponding Al sublattice sites. This partial replacement of Al atoms by Ga acts as a suppressor of other secondary phases and enhances the thermal stability of the tetragonal phase [23]. Moreover, adding Ga to the τ-MnAl phase enhances its intrinsic magnetic moment per unit cell, improves phase stability, and prevents high-temperature decomposition of the vital ferromagnetic phase. This was proven, as we showed that after 800 °C annealing, there is still a large abundance of the τ-MnAl phase in the sample. This suggests that the ultra-fast solidification in the melt allows not only the formation of the precursor phase but also the start of the phase transformation from the initially solidified melt and the ordering of the structure with the incipient formation of phase τ in the precursor ε. Such large values are similar to and even better than the optimal values reported in the literature for MnAl-based alloys. Room-temperature saturation magnetization of 650 kA/m and coercivity of 0.5 T were found in binary MnAl alloys after high-energy ball milling of powders for 120 min [30]. It should be mentioned that stronger milling conditions lead to increases in coercivity but decreases in saturation magnetization. For instance, in [31], MnAlC powder with a tetragonal main phase was hand-ground for 20 h; the *H_c_* of the magnetic powder increased significantly from 0.09 T to 0.36 T, while its *M_s_* decreased from 69.41 Am^2^/kg to 37.1 Am^2^/kg. Processing gallium-doped τ-MnAl powders through ball milling introduces severe plastic deformation and high-anisotropy flake-like particles. A standard 6 h milling process yields a coercivity of 217 kA/m, as reported in [22]. However, in the case of melt-spun MnAl alloys [32], the annealing step successfully modified the magnetic properties of the as-spun ribbons, raising the coercivity from 1.5 to 3.5 kOe, accompanied by an 8% decrease in remanence compared with that of the starting ribbons, achieving 35 Am^2^/kg for cold-rolled and annealed samples. In our work, we obtained a maximum magnetization of about 590 kA/m at room temperature, comparable to higher values obtained in high-energy ball-milled MnAl, a coercive field of about 446 kA/m, or 0.56 T, much higher than reported results on MnAlC hand-ground powders [31], results on Ga-doped MnAl powders [22] or in the case of melt-spun MnAl [32].

## 4. Conclusions

With the aim of optimizing the annealing conditions for achieving good magnetic properties in MnAl alloys with Ga addition, a thorough thermal analysis involving differential scanning calorimetry was performed. Powered by kinetic analysis using two complementary iso-conversional methods, namely, Friedman and Ozawa–Flynn–Wall, the thermal analysis has shown a significant increase in activation energy, indicating a crystallization mechanism that facilitates the formation of the τ-phase from the precursor ε by a single reaction process. The change in the activation energy of crystallization with rising temperature reveals that the overall process combines a thermodynamically driven nucleation barrier and temperature-dependent molecular diffusion upon isothermal annealing. Structural characterization performed using XRD, empowered by full-profile MAUD analysis, has shown that the hexagonal ε phase, predominant in the as-cast sample, transforms massively into the tetragonal τ phase, which becomes predominant at annealing temperatures of about 800 °C. While the hcp ε-phase was found to be predominant in the as-cast samples, after appropriate annealing, it was shown that annealing at 800 °C for 1 h is favorable for continuing ε-τ phase transformation and stabilizing the τ phase. This massive transformation facilitates high remanence values of about 210 kA/m and large coercivities of about 446 kA/m at ambient temperature. These results are consistent with structural findings, as we have shown that after 800 °C annealing, there is still a large abundance of the τ-MnAl phase in the sample. This is more relevant since this large abundance of the τ-MnAl phase, which gives rise to significant magnetic performance, occurs at annealing temperatures that are close to the sintering conditions for producing bulk, compact magnets for real-life applications. These properties will be correlated in further studies involving MnAl alloys with various amounts of Ga added to the binary alloy.

## Figures and Tables

**Table 1:** Maximum temperatures of each exothermic peak, T_peak_, as a function of the heating rate during DSC analysis.

Heating Rate (°C/min)	T_peak_ (°C)
5	442.7 ± 0.1
10	461.6 ± 0.1
15	471.3 ± 0.1
20	472.8 ± 0.1
25	473.8 ± 0.1

**Table 2:** Results obtained from the full-profile MAUD analysis of the diffractogram in Figure 7.

Structural Phases	Lattice Parameters (Å)	Grain Size D (nm)
Rhombohedral phase 6%	a = 12.4828 ± 0.0014 Å c = 7.9120 ± 0.0008 Å	22.73 ± 2 nm
Hexagonal phase 94%	a = 2.6981 ± 0.0002 Å c = 4.3538 ± 0.0004 Å	36.08 ± 4 nm

**Table 3:** Results obtained from the full-profile MAUD analysis of the diffractogram in Figure 8.

Structural Phases	Lattice Parameters (Å)	Grain Size D (nm)
Rhombohedral phase 2%	a = 12.4813 ± 0.0011 Å c = 7.9102 ± 0.0006 Å	37.41 ± 2 nm
Hexagonal phase 27%	a = 2.6962 ± 0.0003 Å c = 4.3577 ± 0.0003 Å	49.72 ± 4 nm
Tetragonal phase 71%	a = 2.7485 ± 0.0002 Å c = 3.5738 ± 0.0004 Å	61.29 ± 4 nm

## Data Availability

The data presented in this study is available on request from the corresponding author. The data is not publicly available due to patenting potential.
